# Magnetic bilayer-skyrmions without skyrmion Hall effect

**DOI:** 10.1038/ncomms10293

**Published:** 2016-01-19

**Authors:** Xichao Zhang, Yan Zhou, Motohiko Ezawa

**Affiliations:** 1 School of Electronic Science and Engineering and Collaborative Innovation Center of Advanced Microstructures, Nanjing University, Nanjing 210093, China; 2 Department of Physics, University of Hong Kong, Pokfulam Road, Hong Kong, China; 3 Department of Applied Physics, University of Tokyo, Hongo 7-3-1, Tokyo 113-8656, Japan

## Abstract

Magnetic skyrmions might be used as information carriers in future advanced memories, logic gates and computing devices. However, there exists an obstacle known as the skyrmion Hall effect (SkHE), that is, the skyrmion trajectories bend away from the driving current direction due to the Magnus force. Consequently, the skyrmions in constricted geometries may be destroyed by touching the sample edges. Here we theoretically propose that the SkHE can be suppressed in the antiferromagnetically exchange-coupled bilayer system, since the Magnus forces in the top and bottom layers are exactly cancelled. We show that such a pair of SkHE-free magnetic skyrmions can be nucleated and be driven by the current-induced torque. Our proposal provides a promising means to move magnetic skyrmions in a perfectly straight trajectory in ultra-dense devices with ultra-fast processing speed.

## Abstract (web summary)

Magnetic skyrmions propagating under an applied current along a nanowire experience the magnus force, deflecting them towards the edges where they may be destroyed, potentially hindering their applications. Here, the authors propose a method to surpass this issue utilizing magnetic bilayers systems.

## Graphical abstract

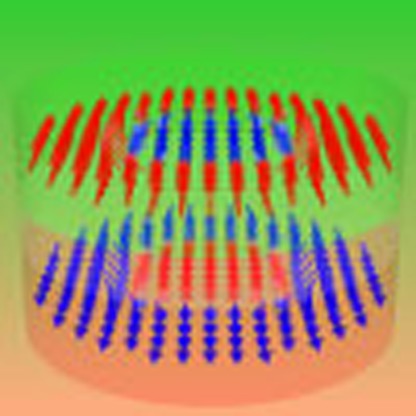

The magnetic skyrmion, a topologically protected spin texture with a quantized topological number^1^,^2^,^3^,^4^,^5^,^6^,^7^,^8^, has been a prominent topic of condensed matter physics since the first experimental observations of skyrmion lattices in bulk non-centrosymmetric magnets^9^,^10^ and thin films^11^. The creation and transmission of an isolated magnetic skyrmion in thin films is a key for future skyrmionics, which utilizes skyrmions as information carriers in advanced memories, logic gates and computing devices^12^,^13^,^14^,^15^. Recently, skyrmions were realized at room temperature^16^,^17^. The strong spin orbit coupling at the interface between the magnetic layer with perpendicular magnetic anisotropy (PMA) and the underlying heavy metal layer provides a sizeable Dzyaloshinskii–Moriya interaction (DMI) to stabilize skyrmions^2^,^18^,^19^,^20^,^21^.

Skyrmions can be displaced by the spin-polarized current, where the angular momentum of the spin-polarized current is transferred from itinerant conduction electrons to the magnetic moment of magnetic skyrmions^2^,^3^,^22^. However, a skyrmion cannot move in a straight line along the driving current direction, as it feels the Magnus force which shifts its trajectory when moving^1^. This is referred to as the Skyrmion Hall effect (SkHE). As a result, magnetic skyrmions may be destroyed at the edges of nanotracks^23^,^24^. Especially, the distance that a skyrmion can be moved drastically reduces as the nanotrack becomes narrower. This will be a roadblock to realize skyrmionic ultradense devices, where it is preferable to use a nanotrack with width of tens of nanometres.

One approach of reducing the SkHE is to explore spin-wave (or magnon)-driven skyrmion motion^25^,^26^,^27^,^28^,^29^,^30^,^31^. It has been shown that a skyrmion can be displaced by magnons induced by thermal gradients in insulating chiral ferromagnets^30^,^31^. It has also been shown that the skyrmion Hall angle may become zero for high-energy magnons^29^. However, as compared with the current-driven skyrmion scheme, it is difficult to generate spin waves in a nanometre-size nanotrack with spectral properties needed for driving the motion of a skyrmion^27^,^32^. It is also difficult to realize a skyrmionic nanocircuit based on thermal gradients. Therefore, we focus on the current-driven skyrmion motion in this work. Here we show that the SkHE is completely suppressed by considering two perpendicularly magnetized ferromagnetic (FM) sublayers strongly coupled via the antiferromagnetic (AFM) exchange interaction with a heavy-metal layer beneath the bottom FM layer^33^,^34^ (Fig. 1a–c). When one skyrmion is created in the top FM layer, another skyrmion is simultaneously created in the bottom FM layer when the interlayer AFM exchange coupling is strong enough. We refer to such a pair of AFM-coupled magnetic skyrmions as a magnetic bilayer-skyrmion (Fig. 1d,e). We show a bilayer-skyrmion can travel over arbitrarily long distances driven by spin currents without touching nanotrack edges thanks to the complete suppression of the SkHE (Fig. 2a,b). Our result provides a guideline for designing realistic ultradense and ultrafast information processing, storage and logic computing devices based on magnetic skyrmions.

## Results

### Hamiltonian

**Figure 1:**
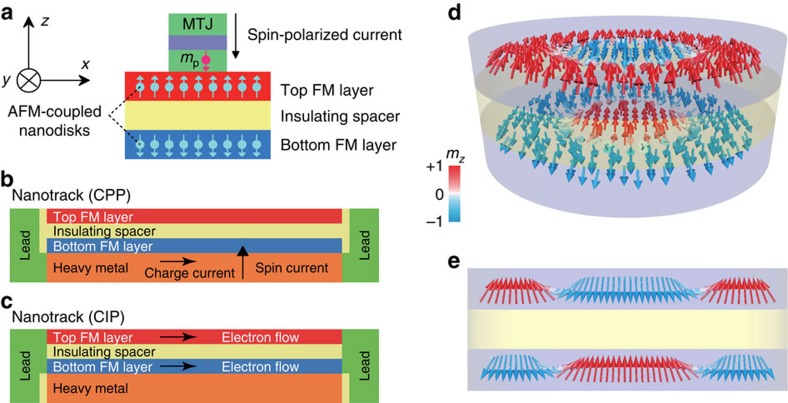
Schematics of the antiferromagnetically exchange-coupled bilayer systems and the bilayer-skyrmion. (**a**) The AFM-coupled bilayer nanodisk with a diameter of 100 nm for bilayer-skyrmion creation. The spin-polarized current (polarization direction **p**=−*z*) is injected into the top ferromagnetic (FM) layer in the central circle with a diameter of 40 nm, which can be realized by means of a magnetic tunnel junction (MTJ) injector above the nanodisk. (**b**) The AFM-coupled bilayer nanotrack (500 × 50 × 3 nm^3^) for the study of the motion of a bilayer-skyrmion driven by the current perpendicular to the plane (CPP). The charge current flows through the heavy-metal substrate along the *x*-direction, which gives rise to a spin current (**p**=+*y*) perpendicularly injected to the bottom FM layer because of the spin Hall effect. The skyrmion in the bottom FM layer is driven by the spin current, whereas the skyrmion in the top FM layer moves accordingly due to the interlayer AFM exchange coupling. (**c**) The AFM-coupled bilayer nanotrack (500 × 50 × 3 nm^3^) for the study of the motion of a bilayer-skyrmion driven by the in-plane current (CIP). The electrons flow towards the right in both the top and bottom FM layers, that is, the corresponding charge currents flow along the −*x* direction. The skyrmions in both the top and bottom FM layers are driven by the current. In all the models, the thickness of both the top FM layer, the bottom FM layer and the insulating spacer are equal to 1 nm. The initial state of the top FM layer is almost spin-up (pointing along +*z*) and that of the bottom FM layer is almost spin-down (pointing along −*z*). (**d**) Illustration of a pair of skyrmions, (that is, the bilayer-skyrmion) in an AFM-coupled nanodisk. (**e**) Side view of the bilayer-skyrmion along the diameter of **d**. The colour scale represents the out-of-plane component of the magnetization, which is used throughout this paper.

We investigate a bilayer system where the top and bottom FM layers are coupled antiferromagnetically by the exchange interaction, as illustrated in Fig. 1a. The Hamiltonian for each layer reads


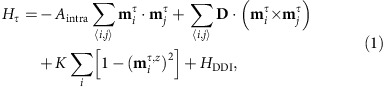


where *τ* is the layer index (*τ*=T, B), 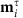 represents the local magnetic moment orientation normalized as 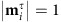 at the site *i*, and 〈*i*,*j*〉 runs over all the nearest neighbour sites in each layer. The first term represents the FM exchange interaction with FM exchange stiffness *A*_intra_. The second term represents the DMI with the DMI vector **D**. The third term represents the PMA with the anisotropic constant *K*. *H*_DDI_ represents the dipole–dipole interaction. There exists an AFM exchange coupling between the top and bottom FM layers,


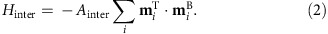


The sign of *A*_inter_ is negative for the interlayer AFM interaction. We assume that the spins in the top FM layer are pointing upward. Then the spins in the bottom FM layer are pointing downward because of the interlayer AFM exchange coupling.

### Bilayer skyrmions

The Hamiltonian equation (1) allows the excitation of a spin texture carrying the topological number (the skyrmion number) *Q*_*τ*_ in each FM layer. Spins swirl continuously around the core and approach the ground-state value asymptotically in the skyrmion spin texture. The skyrmion numbers of the top and bottom FM layers are quantized, and opposite since all the spins are inverted. We choose the convention that *Q*_T_=−*Q*_B_=1.

A skyrmion is characterized by its topological stability in each FM layer. Even if the skyrmion spin texture is deformed continuously, its skyrmion number cannot change as it is quantized. It can be neither destroyed nor separated into pieces, that is to say, a skyrmion is topologically protected.

When the AFM interaction equation (2) is taken into account, the skyrmions in the two layers are bound. Such a pair of skyrmions may be called a bilayer-skyrmion. We estimate the excitation energy based on the Hamiltonian *H*_total_=*H*_T_+*H*_B_+*H*_inter_. By determining numerically the spin profile of each skyrmion, we compare the energy of one bilayer-skyrmion state with the energy of the homogeneous state in Fig. 3c as a function of the DMI. The energy of the bilayer-skyrmion becomes lower than that of the uniform ground state for *D*≳4 mJ m^−2^ in a typical sample, which is the threshold value. When the DMI strength is much smaller than the threshold value, it is hard to create a bilayer-skyrmion. On the other hand, when it is much larger, a deformed bilayer-skyrmion is generated. As a result, it is preferable to tune the DMI strength around the threshold value to create an isolated bilayer-skyrmion.

### Skyrmion Hall effect

To understand the origin of the SkHE, we analyse the dynamics of a skyrmion based on the Thiele equation. The Thiele equation describes the centre-of-mass motion of a skyrmion without affecting its form in each FM layer^3^,^22^,^35^,^36^,^37^,^38^.

Suppressing the layer index, the Thiele equation for the skyrmion driven by the in-plane current (CIP) reads,


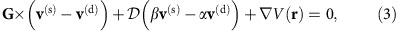


in each FM layer. Here, **v**^(d)^=**R** is the drift velocity; **v**^(s)^ is the velocity induced by spin-polarized current; **G**=(0,0,*G*) is the gyromagnetic coupling vector, where *G*=4*πQ* with the skyrmion number *Q*; 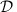 represents the dissipative force; *V*(**r**) is the confining potential induced by the sample edges; *α*≃0.1 is the Gilbert damping constant and *β* is the strength of the non-adiabatic torque. The first term on the left hand side of equation (3) corresponds to the Magnus force. We consider a skyrmion moving along the *x* axis far away from the edge. Thus, we set 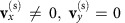 and *V*=0, to find


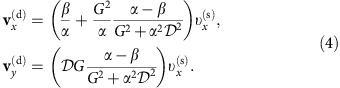


Consequently, a skyrmion undergoes a transverse motion 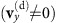 because it carries a non-zero skyrmion number (*G*≠0).

Similarly, the Thiele equation for the skyrmion driven by the current perpendicular to the plane (CPP) reads^36^,


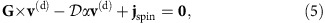


where **j**_spin_ is the spin current induced by the charge current in the heavy-metal layer. The first term on the left hand side of equation (5) corresponds to the Magnus force. We find


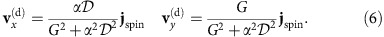


A skyrmion undergoes a transverse motion 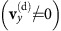 for *G*≠0, similar to the CIP case.

It is concluded that a moving skyrmion feels the Magnus force and bends towards the direction perpendicular to the driving current. The direction of the Magnus force is opposite when the sign of the skyrmion number *Q* is opposite. For instance, if a skyrmion moves along the +*x* direction it will be bent towards the +*y* (−*y*) direction in the top (bottom) FM layer.

A skyrmion is protected topologically only when it is far away from the edge. When the skyrmion touches an edge, the boundary condition is violated and the topological stability is lost. The skyrmion number *Q* is no longer quantized and may change continuously. We present a typical example of the annihilation process of the skyrmion because of the SkHE in Supplementary Figs 1 and 2 and Supplementary Movies 1 and 2. It is found by micromagnetic simulations that a skyrmion can only travel 2 nm along the nanotrack at a driving current density of 1 × 10^10^ A m^−2^ when the nanotrack width is reduced to 24 nm for typical Co|Pt material parameters.

### Motion of a bilayer-skyrmion in an AFM-coupled nanotrack

**Figure 2:**
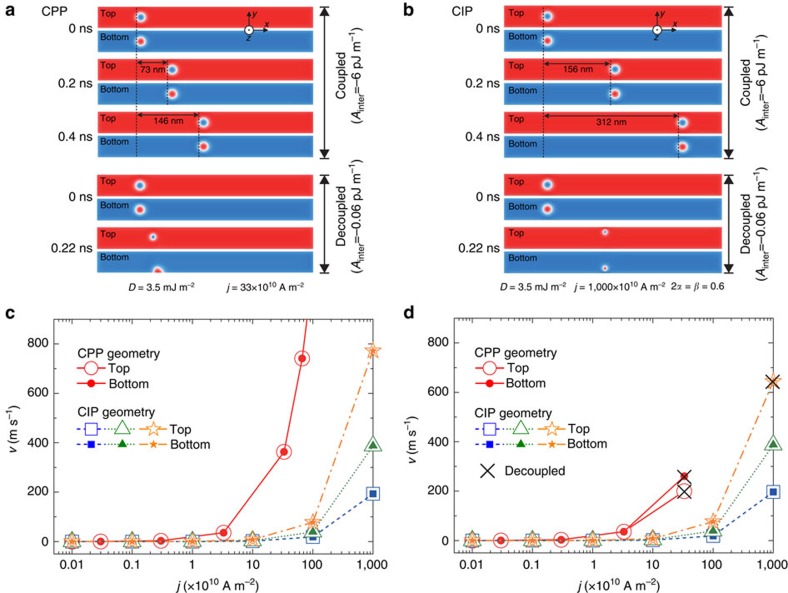
Current-induced motion of skyrmions in the top and bottom FM layers of an AFM-coupled bilayer nanotrack. Top views of the motion of skyrmions at selected interlayer exchange coupling constants and times driven by spin currents with (**a**) the current-perpendicular-to-plane (CPP) injection geometry and (**b**) the current-in-plane (CIP) injection geometry. The size of the AFM-coupled bilayer nanotrack is 500 × 50 × 3 nm^3^, where *D*=3.5 mJ m^−2^. The skyrmions are initially created by the MTJ skyrmion injector placed on the top FM layer at *x*=100 nm. For the CPP case, the spin current in the bottom FM layer is applied along +*z* but polarized along +*y*. The skyrmion in the bottom FM layer moves along the nanotrack driven by the spin current, whereas the skyrmion in the top FM layer moves accordingly due to the interlayer AFM exchange coupling. For the CIP case, the skyrmions in both the top and bottom FM layers are driven by in-plane spin-polarized currents. The skyrmions velocities in the top (open symbols) and bottom (solid symbols) FM layers as functions of the current density *j* for (**c**) the large interlayer AFM exchange stiffness *A*_inter_=−6 pJ m^−1^ and (**d**) the small interlayer AFM exchange stiffness *A*_inter_=−0.06 pJ m^−1^. For the CIP case, the square, triangle and star symbols indicate different values of the non-adiabatic torque constant *β* of 0.15, 0.3 and 0.6, respectively. The cross symbol denotes the decoupling as well as the destruction of skyrmions in the top and bottom FM layers because of a large driving current density and a small interlayer AFM exchange coupling, where the velocities are calculated just before the destruction of skyrmions.

The skyrmions in the two FM layers of the bilayer system move simultaneously when they are strongly bound by the interlayer AFM exchange coupling. As a result, the skyrmion in the top FM layer follows the motion of the skyrmion in the bottom FM layer even when the current is injected only into the bottom FM layer. There is no SkHE for a bilayer-skyrmion with sufficient interlayer AFM coupling strength. This is confirmed by micromagnetic simulations in Fig. 2a,b. When the interlayer AFM exchange coupling is strong enough, two skyrmions are tightly bound and the Magnus forces acting on the skyrmions between the top and bottom FM layers are exactly cancelled. Accordingly, the bilayer-skyrmion moves in a straight line along the nanotrack for sufficiently strong AFM exchange coupling (see Supplementary Movies 3 and 4 for details). However, for zero or sufficiently small interlayer AFM exchange coupling, a skyrmion in the top FM layer moves left-handed and the skyrmion in the bottom FM layer moves right-handed (see Supplementary Movies 5,6,8 for details). Once the skyrmions in the top and bottom FM layers are decoupled, the SkHE becomes active, leading to the destruction of skyrmions in the top FM layer and/or bottom FM layer by touching the nanotrack edges as shown in the decoupled case of Fig. 2a,b.

The relation between the magnitude of the injected current and the bilayer-skyrmion velocity is shown in Fig. 2c,d. There are some prominent features as shown in Fig. 2c,d. First, the velocity is proportional to the injected current density, following the *j*–*v* relation of a conventional single skyrmion. The CPP scheme has been proven to be much more efficient to drive the skyrmions than the CIP method, consistent with the reported results of isolated skyrmions^2^,^3^. Second, for weak coupling case in Fig. 2d (here *A*_inter_=−0.06 pJ m^−1^), the bilayer-skyrmion still moves as a whole for small and medium current densities and only decoupled and destroyed because of the SkHE for large current density. This is because the SkHE increases as the current increases, which acts as the repulsive force between two skyrmions. Third and most important, the AFM-coupled bilayer-skyrmion can move at a high speed of a few hundreds to a few thousands metres per second without a transverse shift with almost the same low-current density threshold feature as a single skyrmion in typical Co|Pt material. This can be seen from the low-current regime of Fig. 2c,d (cf. ref. 3) and the comparison with the single skyrmion as given in Supplementary Tables 1 and 2.

These results demonstrate that the bilayer-skyrmion may possess multiple advantages such as enhanced *j*–*v* slope especially at higher driving current density, small depinning threshold current density (see Supplementary Tables 1 and 2 for details) and maximum speed of up to ∼1,000 m s^−1^. It is noted that for slightly different material parameters, similar conclusions remain as for the Co|Pt system.

### Creation of a bilayer-skyrmion by vertical spin current

We proceed to discuss how to create a bilayer-skyrmion. We propose two different ways. In the first method, we inject the spin-polarized current (polarized along −*z*) perpendicularly into the central circle region of the top FM layer, as illustrated in Fig. 1a.

**Figure 3:**
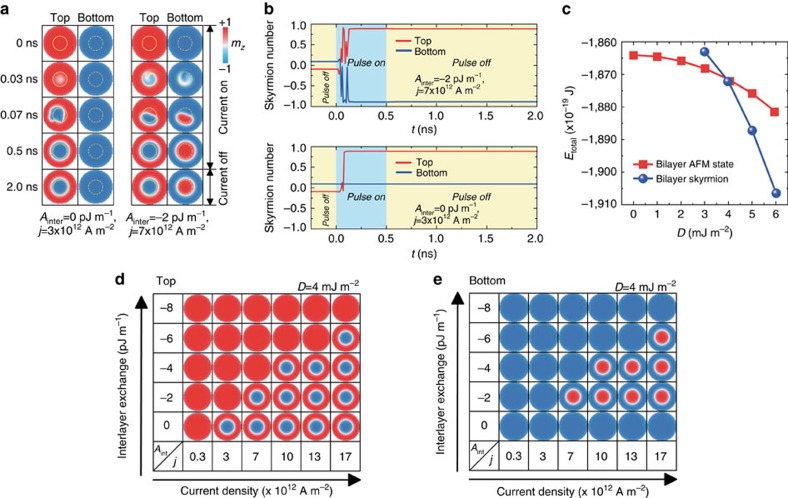
Creation of skyrmions in the AFM-coupled bilayer nanodisk and time evolution of the skyrmion number. (**a**) Injection of skyrmions in the AFM-coupled bilayer nanodisk (*D*=4 mJ m^−2^) with/without the interlayer AFM exchange coupling. A 500-ps-long spin-polarized current is injected into the top FM layer followed by a 1,500-ps-long relaxation. The spin-polarized current injection region in the top FM layer and the corresponding region in the bottom FM layer are denoted by solid and dash yellow circles, respectively. The interlayer AFM exchange coupling constant *A*_inter_ is set as 0 or −2 pJ m^−1^, whereas the corresponding interface AFM exchange coupling constant *σ* equals to 0 or −2 mJ m^−2^. (**b**) The evolution of the skyrmion number of the top and bottom FM layers in the nucleation process of skyrmions corresponding to **a**. (**c**) Total micromagnetic energy *E*_total_ (including the intralayer exchange, interlayer exchange, dipole–dipole, anisotropy and DMI energy) for a bilayer-skyrmion and the AFM-coupled ground state as a function of the DMI constant *D*. Relaxed state of (**d**) the top FM layer and (**e**) the bottom FM layer after the injection of a 500-ps-long spin-polarized current for various current density *j* and interlayer AFM exchange coupling constant *A*_inter_.

We demonstrate how the spin textures develop and evolve in Fig. 3a. The spins start to flip in both FM layers following the spin current injection only in the top FM layer. When there is no interlayer AFM exchange coupling, a skyrmion is formed only in the top FM layer (see Supplementary Movie 9 for details). By contrast, a skyrmion is formed accordingly in the bottom FM layer upon the current injection in the presence of the interlayer AFM exchange coupling (see Supplementary Movie 10 for details).

Figure 3b shows the evolution of the skyrmion number corresponding to Fig. 3a. In the process of creating a bilayer-skyrmion, the skyrmion number of both top and bottom FM layers oscillate at the initial stage for *t*<0.2 ns, and rapidly increases to ±1. The skyrmion numbers remain stable even when the current is switched off, demonstrating that the bilayer-skyrmion is topologically protected. During this process, the spins in the top and bottom FM layers are always anti-parallel.

A comment is in order. The saturated skyrmion number is not exactly *Q*=1 but *Q*=0.89, because there is a background skyrmion-number contribution of −0.09 from the tilting edge spins. Accordingly, the calibrated skyrmion number is *Q*=0.98, which is almost unity within numerical accuracy.

We present a nucleation phase diagram of a bilayer-skyrmion as functions of the current density and the interlayer AFM exchange coupling in Fig. 3d,e. When the magnitude of the injected current is strong enough, the bilayer-skyrmion is created. This is due to the fact that spin flip costs a certain energy. On the other hand, if the interlayer AFM exchange coupling is too strong, the bilayer-skyrmion is suppressed because of the fact that the nucleation field and the coercivity of the top and bottom FM layers increase with the interlayer AFM exchange coupling, leading to a larger threshold current density for nucleation.

### Creation of a bilayer-skyrmion from a bilayer-domain wall pair

A skyrmion can also be created from a domain wall (DW) pair by using a junction geometry^13^,^14^. In this scenario, we first create an AFM-coupled DW pair within an AFM-coupled bilayer nanotrack, where a local CPP injection with −*z* direction is applied on the top FM layer. We show how the spins start to flip in the top FM layer and subsequently in the bottom FM layer driven by the AFM exchange coupling force in Supplementary Fig. 3a. Then, the bilayer DW pair is shifted by applying the CPP injection, as shown in the process from *t*=50 ps to *t*=120 ps in Supplementary Fig. 3b. Here we consider the vertical injection of a spin current towards +*z* and polarized along+*y* in the bottom FM layer. The CPP injection moves the bilayer-DW along the nanotrack. When the bilayer-DW arrives at the junction interface (*t*=170 ps), both the end spins of the DW are pinned at the junction, whereas the central part of the DW continues to move because of spin-transfer torques (STTs) in the wide part of the nanotrack. Therefore, the structure is deformed into a curved shape and a bilayer-skyrmion texture forms at *t*=190 ps (see Supplementary Fig. 3 and Supplementary Movie 11 for details).

### Consideration for experiments

There are already some experimental ways to host and manipulate DWs in systems similar to the proposed device in this work. A prototypical example based on the three-dimensional implementation of the racetrack memory architecture is the Co|Ni multilayers with strong PMA separated by an AFM layer (for example, Ru, Cr and so on). Such devices can be fabricated by atomic layer deposition and patterned with Electron Beam Lithography and ion milling^18^,^33^,^39^. Strong PMA is derived from the spin-orbit coupling at the interface of the heavy metal (for example, Pt) and FM material (Co in this case). Various material properties such as the total *M*_S_ and the interfacial coupling of the synthetic antiferromagnetic layers can be controlled within the reach of leading-edge material fabrication and deposition techniques^18^,^33^. It is very likely that interfacial DMI might be present and controlled by atomically engineering the interface and material composition. Therefore, it is promising to engineer a number of materials and design properties such as the interfacial DMI (in the range of 0.1∼10 mJ m^−2^) and the interlayer AFM exchange coupling (−0.1∼−10 pJ m^−1^) to host bilayer-skyrmions^16^,^18^,^33^,^40^.

## Discussion

The relation between the velocity and the injected current for a bilayer-skyrmion is the same as that for a monolayer-skyrmion, as shown in Fig. 2c,d. The critical current density required to drive the bilayer-skyrmion into motion in the CPP case equals ∼0.01 × 10^10^ A m^−2^, at which the speed of the bilayer-skyrmion is ∼0.15 m s^−1^. While in the CIP case, the critical current required to drive the skyrmion is approximately ten times larger than that of the CPP case. The critical current density is in good agreement with the value of a single isolated skyrmion in the monolayer nanotrack shown in the previous analysis^3^, indicating that the interlayer AFM exchange coupling between the top and bottom skyrmions does not increase the depinning current of the skyrmions (see Supplementary Tables 1 and 2 for details).

It is also worth noting that the interlayer AFM exchange coupling does not produce a mass of the bilayer-skyrmion. When the driving current is suddenly turned off, the bilayer-skyrmion stops high-speed motion immediately (see Supplementary Movie 12 for details).

We also investigate the case where the DMI constant *D* is different between the top and bottom FM layers. It is found that the results of the current-driven motion of a bilayer-skyrmion do not change much as the DMI only changes the size of the skyrmion (see Supplementary Movies 13 and 14 for details). The massless property and its robustness make the bilayer-skyrmion an ideal candidate for practical applications.

The maximum velocity of a monolayer-skyrmion is typically much less than 10^2^ m s^−1^, limited by the edge confining force ∼*D*^2^/*J* (see ref. 41 for details). Remarkably, the maximum velocity of a bilayer-skyrmion is much larger than a monolayer-skyrmion. The bilayer-skyrmion can move along the central line of the nanotrack at a high speed of a few hundred metres per second.

In summary, we have presented a solution of inhibiting the Hall effect of skyrmions without affecting the topological protection, by exploring a device comprised of antiferromagnetically exchange-coupled bilayer nanodisks and nanotracks. Compared with the mostly investigated skyrmion in the monolayer system, the bilayer-skyrmion exhibits distinct characteristics with regard to the current-transport behaviour and robustness. First, it can move strictly along the direction of the spin-polarized current flow, which is necessary for ultradense memory applications. This is in high contrast with the case of the monolayer-skyrmion, where the skyrmion is destroyed by the edges of the nanotracks. Second, it will be robust against magnetic field perturbations, which might be generated externally or internally within the device circuitry, as the net magnetic moment of the bilayer-skyrmion is zero. Our proposal of transporting skyrmion for arbitrarily long distances at much enhanced velocity may be useful for versatile applications such as ultradense memory and ultrafast information processing. Similar ideas can be extended to a multilayer or a superlattice where the skyrmions are strongly coupled to realize a better manipulation of skyrmions in confined geometries.

## Methods

### Landau–Lifshitz–Gilbert (LLG) equation for CPP

We may apply the spin-polarized CPP injection from the magnetic tunnel junction or the spin Hall effect in heavy-metal layer^3^,^15^,^24^,^36^,^42^. For the CPP case, the spin current is perpendicular to the film plane, whereas the charge current can be either in the film plane such as the spin Hall effect scenario or perpendicular to the film plane such as the perpendicular magnetoresistive random access memory (MRAM) where a perpendicularly magnetized polarizer is incorporated. The dynamics of the magnetization **m**_*i*_ at the lattice site *i* is governed by the LLG equation. In the case of the CPP injection, we numerically solve the LLG equation extended into the following form,


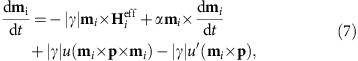


with the layer index suppressed. Here, 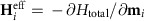 is the effective magnetic field induced by the Hamiltonian *H*_total_=*H*_T_+*H*_B_+*H*_inter_; *γ* is the gyromagnetic ratio; *α* is the Gilbert damping coefficient originating from the spin relaxation; *u* is the STT coefficient; *u*′ is the field-like out-of-plane STT coefficient and **p** represents the electron polarization direction. We have 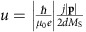 with *μ*_0_ the vacuum magnetic permittivity, *d* the film thickness of FM layer, *M*_S_ the saturation magnetization and *j* the current density. We take −*z* direction of the spin polarization for creating the skyrmion, whereas +*y* direction of the spin polarization for moving the skyrmion. The STT is induced either by injection from a magnetic tunnel junction polarizer or by the spin Hall effect^3^,^36^,^42^.

### LLG equation for CIP

For the CIP case, the current is applied along the nanotrack axial direction^3^,^36^,^42^. We numerically solve the LLG equation with the addition of the STT term in the following form,


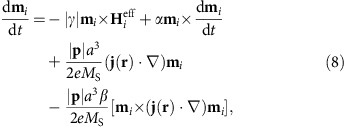


where *β* is the strength of the non-adiabatic torque and *a* is the lattice constant.

### Micromagnetic modelling

The three-dimensional micromagnetic simulations are performed using the well-established Object Oriented MicroMagnetic Framework developed at the National Institute of Standards and Technology^43^. Additional extension module is employed to include the DMI in the simulation^2^,^3^,^44^. The time-dependent magnetization dynamics is governed by the LLG equation including both antidamping-like and field-like STTs^43^,^45^,^46^,^47^,^48^. The average energy density *E* is a function of vectorial magnetization **M**, which includes the intralayer exchange, the interlayer exchange, the anisotropy, the applied field (Zeeman), the demagnetization (dipole–dipole) and the DMI energy terms. For micromagnetic simulations, the standard intrinsic magnetic parameters are adopted from refs 2, 3: Gilbert damping coefficient *α*=0.3, gyromagnetic ratio *γ*=−2.211 × 10^5^ m A^−1^ s^−1^, saturation magnetization *M*_S_=580 kA m^−1^, intralayer exchange stiffness *A*_intra_=15 pJ m^−1^, DMI constant *D*=0∼6 mJ m^−2^ and PMA *K*=0.8 MJ m^−3^ unless otherwise specified. The interlayer exchange coefficient *A*_inter_ is set from 0 to −10 pJ m^−1^, whereas the corresponding interface exchange coefficient *σ* equals from 0 to −10 mJ m^−2^ (*σ*=*A*_inter_/1 nm in our case). The negative value of the constant *σ* denotes that the interface is antiferromagnetically exchange-coupled. The field-like out-of-plane STT coefficient *u*′ is set to zero, and the Oersted field is neglected for simplicity. The polarization rate (|**p**|) of the spin current used in all simulations is fixed at 0.4. All the samples are discretized into tetragonal volume elements with the size of 2 × 2 × 1 nm^3^ in the simulation, which is sufficiently smaller than the typical exchange length (∼4.3 nm) as well as the skyrmion size, ensuring a balance between numerical accuracy and computational efficiency. For all simulation of current-driven skyrmions reported throughout this paper, the skyrmions are first created at the designed spot of the nanotrack (*x*=100 nm) by a local spin CPP of the top FM layer. Then the system is relaxed to an energy minimum state without applying any current. Next, we start the timer and the spin current is injected into the nanotrack in the CIP or CPP scheme as shown in Fig. 1. For the configuration of CIP, the electrons flow towards the right in both the top and bottom FM layers, that is, the charge currents flow towards the left, whereas for the configuration of CPP, the electrons flow upward along +*z* only in the bottom FM layer.

## Additional information

**How to cite this article:** Zhang, X. *et al*. Magnetic bilayer-skyrmions without skyrmion Hall effect. *Nat. Commun.* 7:10293 doi: 10.1038/ncomms10293 (2016).

## Supplementary Material

Supplementary Information Supplementary Figures 1-3 and Supplementary Tables 1-2

Supplementary Movie 1 Destruction of a monolayer-skyrmion in a nanotrack.

Supplementary Movie 2 Close view of the destruction process of the monolayer-skyrmion by touching the edge.

Supplementary Movie 3 Motion of skyrmions with a large interlayer AFM exchange coefficient driven by the perpendicular-to-plane current.

Supplementary Movie 4 Motion of skyrmions with a large interlayer AFM exchange coefficient driven by the in-plane current.

Supplementary Movie 5 Motion of skyrmions with a small interlayer AFM exchange coefficient driven by the perpendicular-to-plane current.

Supplementary Movie 6 Motion of skyrmions with a small interlayer AFM exchange coefficient driven by the in-plane current.

Supplementary Movie 7 Motion of skyrmions with zero interlayer exchange coefficient driven by the perpendicular-to-plane current.

Supplementary Movie 8 Motion of skyrmions with zero interlayer exchange coefficient driven by the in-plane current

Supplementary Movie 9 Nucleation process of a bilayer-skyrmion without AFM exchange coupling.

Supplementary Movie 10 Nucleation process of a bilayer skyrmion with AFM exchange coupling.

Supplementary Movie 11 Nucleation of skyrmions from DW pairs driven by vertical current in the AFM-coupled bilayer nanotrack.

Supplementary Movie 12 Sudden stop of a bilayer-skyrmion driven by the perpendicular-to-plane current.

Supplementary Movie 13 Motion of a bilayer-skyrmion with different DMI in the top and bottom FM layers driven by the perpendicular-to-plane current.

Supplementary Movie 14 Motion of a bilayer-skyrmion with different DMI in the top and bottom FM layers driven by the in-plane current.
