# Supplementary material for: Magnetic bilayer-skyrmions without skyrmion Hall effect
Source: Nat Commun. 2016 Jan 19;7:10293. doi: 10.1038/ncomms10293 (PMC4735649; doi:10.1038/ncomms10293)
Supplement: Supplementary Information — Supplementary Figures 1-3 and Supplementary Tables 1-2 [file ncomms10293-s1.pdf]

Supplementary Figures

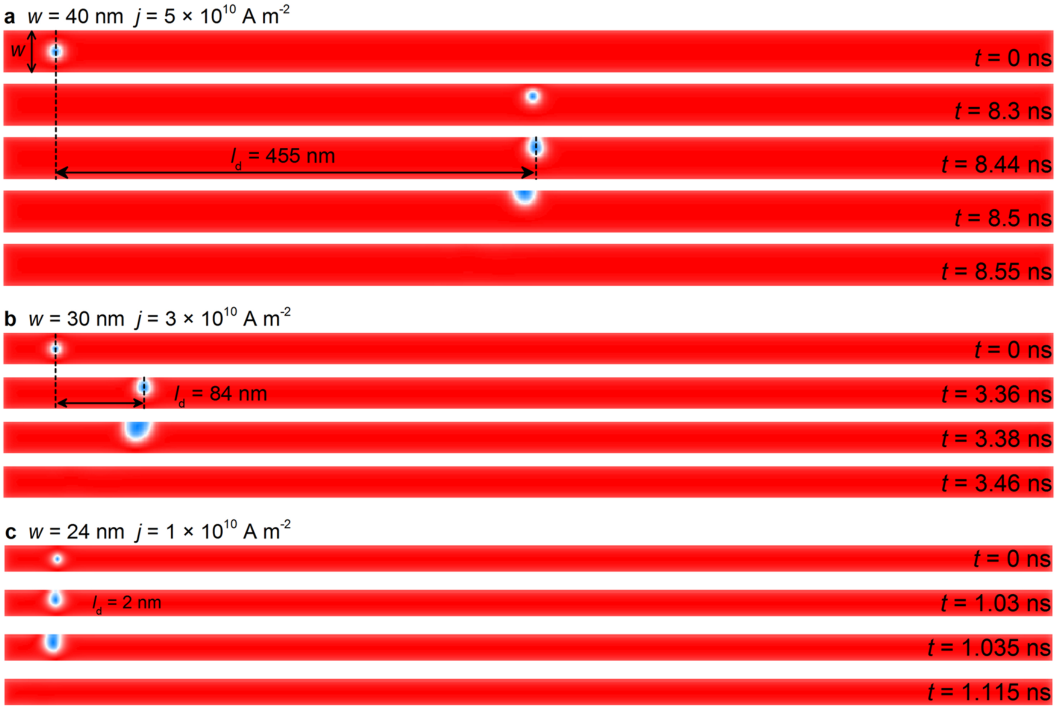

Supplementary Figure 1 | Motion and destruction of a monolayer skyrmion driven by the CPP current in nanotracks with the different track width *w* and the current density *j*. The skyrmion is driven by the CPP current in the monolayer nanotrack (1000 nm × *w* nm × 0.4 nm). The spin current is applied along +*z* but polarized along +*y*, which could be realized by the spin Hall effect. l_d represents the horizontal distance between the position where the skyrmion is destroyed and the initial position of the skyrmion. When the width of the nanotrack equals 40 nm, the CPP current-driven skyrmion is destroyed due to the skyrmion Hall effect (SkHE) when it moves about 460 m at the current density of 5 × 10^{10} A m^{-2} (see Supplementary Movie 1). The velocity of the skyrmion before its destruction equals ~ 55 m s^{-1}. When the width of the nanotrack reduces to 30 nm, the skyrmion is destroyed *en route* due to the SkHE at a smaller driving current density of 3 × 10^{10} A m^{-2}, when moves ~ 80 nm. When the nanotrack width is further decreased to 24 nm, the skyrmion is destroyed shortly with a displacement of 2 nm when the driving current of 1 × 10^{10} A m^{-2} is turned on. The color denotes the out-of-plane component of the magnetization, i.e., blue for spins pointing into the plane, red for spins pointing out of the plane, white for spins pointing in the film plane (similar hereinafter).

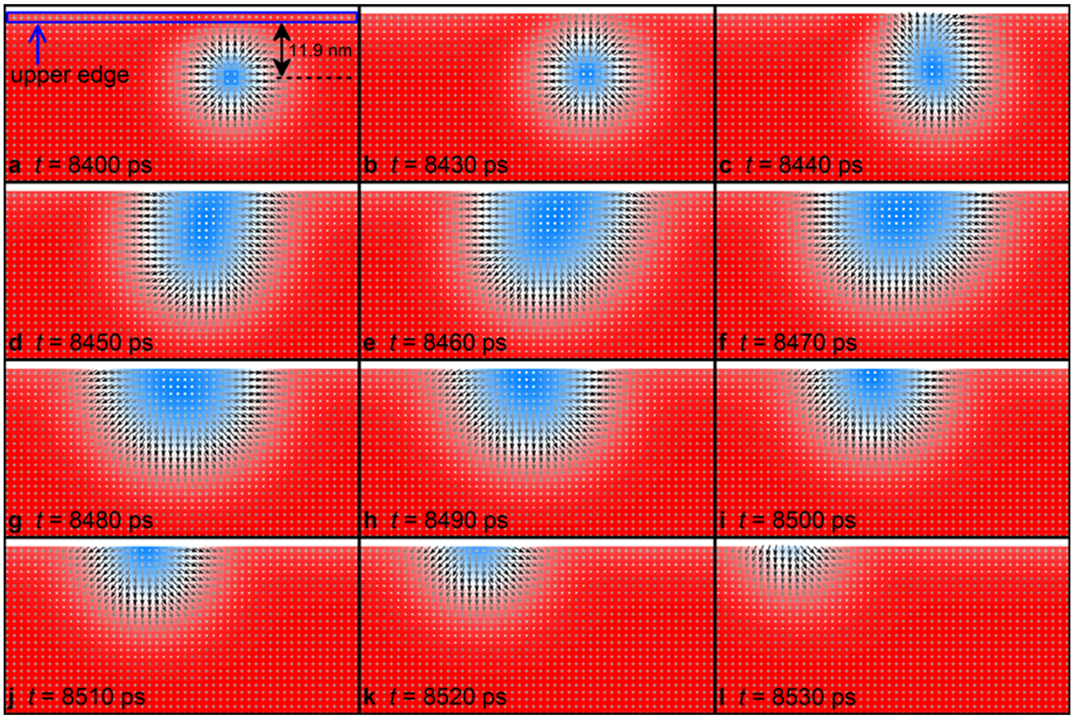

Supplementary Figure 2 | Top views of the destruction of a monolayer-skyrmion due to the skyrmion Hall effect at selected times. The monolayer-skyrmion is driven by a spin current ($j = 5 \times 10^{10}$ A m^{-2}) with the CPP geometry in a nanotrack. The size of the nanotrack is 1000 nm × 40 nm × 0.4 nm, where $D = 3.0$ mJ m^{-2}. The skyrmion is initially created by the MTJ skyrmion injector placed on top of the monolayer nanotrack at $x = 50$ nm, where the distance between the skyrmion core and the upper edge equals 20 nm. The spin current flows along $+z$ (polarized along $+y$). At (**a**) t = 8400 ps, the skyrmion moves along the monolayer nanotrack about a few hundreds of nanometers but the distance between the skyrmion core and the upper edge decreases to only 12 nm due to the SkHE. When $t > 8400$ ps as shown in (**b**)-(**l**), the distance is reduced to less than 12 nm. The magnetization at the upper edge of the skyrmion starts to rotate and an instability starts to develop, which breaks the topological protection of the skyrmion and leads to the annihilation of the skyrmion at the thin film edge. See also Supplementary Movie 2.

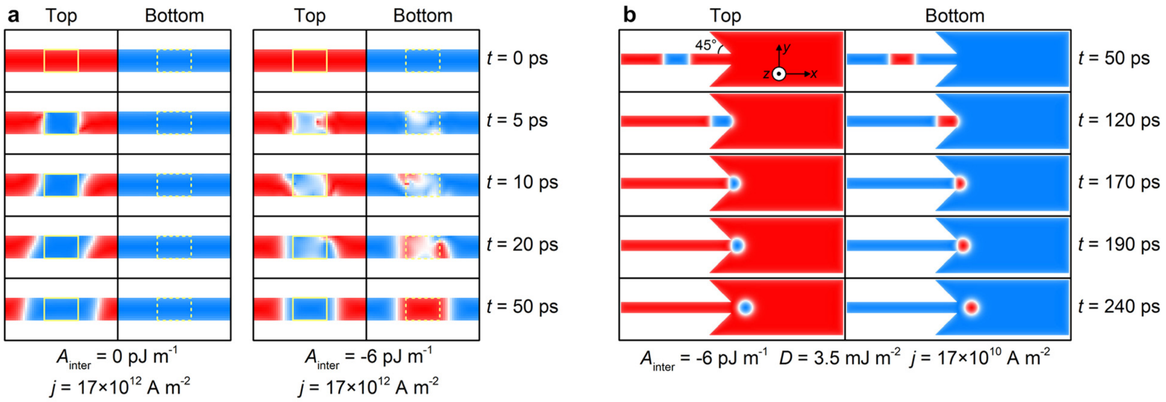

Supplementary Figure 3 | Creation of a bilayer-DW pair, its conversion into a bilayer-skyrmion, and their motions driven by vertical current in an AFM-coupled bilayer nanotrack. The length, wide width and narrow width of the nanotrack (D = 3.5 mJ m^{-2}) equal 400 nm, 100 nm, and 20 nm, respectively. (**a**) A local vertical spin current (j = 17 × 10^{12} A m^{-2}, **p** = -z) is perpendicularly applied to the top FM layer of the narrow side (85 nm < x < 115 nm) before t = 50 ps. When the top and bottom FM layers are decoupled (A_{inter} = 0 pJ m^{-1}), only one DW pair is generated in the top FM layer. However, when the top and bottom FM layers are coupled (A_{inter} = -6 pJ m^{-1}), a bilayer-DW pair is created at t = 50 ps. (**b**) A global vertical spin current is perpendicularly applied to the bottom FM layer (towards +z, **p** = +y) when the bilayer-DW pair is created at t = 50 ps. The current density j in the wide part equals 1.7 × 10^{11} A m^{-2}, which is proportional to that inside the narrow part with respect to the ratio of the narrow width to the wide width. The 45-dgree notches are employed to reduce the required current density for the DW-skyrmion conversion. When the global driving current is turned on at t = 50 ps, the local DW injection current is turned off at the same time. See also Supplementary Movie 11.

Supplementary Tables

Supplementary Table 1 | States of the monolayer-skyrmions and bilayer-skyrmions driven by the in-plane current at low current densities in nanotracks. The interlayer exchange coefficient A_{inter} of the bilayer-skyrmions changes from -0.06 pJ m^{-2} to -6 pJ m^{-2}. In the configuration of current-in-plane (CIP) geometry, the electrons flow toward the right in both the top and bottom FM layers, i.e., the charge currents flow toward the left. In this case, the interlayer antiferromagnetic exchange coupling in the bilayer-skyrmions system has no impact on the depinning current density of the skyrmion motion, which indicates the same spin-torque-driven mechanism for both the monolayer and bilayer skyrmions.

	A_{inter} (pJ m^{-2})	$j = 5 \times 10^8$ A m^{-2}	$j = 5 \times 10^9$ A m^{-2}
Monolayer Skyrmion		Pinned	Depinned
Bilayer Skyrmion (Top Layer)	-0.06	Pinned	Depinned
Bilayer Skyrmion (Bottom Layer)		Pinned	Depinned
Bilayer Skyrmion (Top Layer)	-0.60	Pinned	Depinned
Bilayer Skyrmion (Bottom Layer)		Pinned	Depinned
Bilayer Skyrmion (Top Layer)	-6.00	Pinned	Depinned
Bilayer Skyrmion (Bottom Layer)		Pinned	Depinned

Supplementary Table 2 | States of the monolayer-skyrmions and bilayer-skyrmions driven by the perpendicular-to-plane current at low current densities in nanotracks. The interlayer exchange coefficient A_{inter} of the bilayer-skyrmions changes from -0.06 pJ m^{-2} to -6 pJ m^{-2}. In the configuration of current-perpendicular-to-plane (CPP) geometry, the electrons flow toward the top only in the bottom FM layer. In this case, the depinning current density of the antiferromagnetically exchange-coupled bilayer-skyrmions is basically at the same order of magnitude as that of the monolayer-skyrmions. It should be noted that velocity of the monolayer and bilayer skyrmions are very small at the depinning current densities.

	A_{inter} (pJ m^{-2})	$j = 5 \times 10^7$ A m^{-2}	$j = 5 \times 10^8$ A m^{-2}
Monolayer Skyrmion		Pinned	Depinned
Bilayer Skyrmion (Top Layer)	-0.06	Pinned	Depinned
Bilayer Skyrmion (Bottom Layer)		Pinned	Depinned
Bilayer Skyrmion (Top Layer)	-0.60	Pinned	Depinned
Bilayer Skyrmion (Bottom Layer)		Pinned	Depinned
Bilayer Skyrmion (Top Layer)	-6.00	Pinned	Depinned
Bilayer Skyrmion (Bottom Layer)		Pinned	Depinned
